# High resolution global gridded data for use in population studies

**DOI:** 10.1038/sdata.2017.1

**Published:** 2017-01-31

**Authors:** Christopher T. Lloyd, Alessandro Sorichetta, Andrew J. Tatem

**Affiliations:** 1 WorldPop, Geography and Environment, University of Southampton, Highfield Campus, Southampton SO17 1BJ, UK; 2 Flowminder Foundation, Roslagsgatan 17, Stockholm SE-11355, Sweden

**Keywords:** Developing world, Geography, Environmental social sciences, Research data

## Abstract

Recent years have seen substantial growth in openly available satellite and other geospatial data layers, which represent a range of metrics relevant to global human population mapping at fine spatial scales. The specifications of such data differ widely and therefore the harmonisation of data layers is a prerequisite to constructing detailed and contemporary spatial datasets which accurately describe population distributions. Such datasets are vital to measure impacts of population growth, monitor change, and plan interventions. To this end the WorldPop Project has produced an open access archive of 3 and 30 arc-second resolution gridded data. Four tiled raster datasets form the basis of the archive: (i) Viewfinder Panoramas topography clipped to Global ADMinistrative area (GADM) coastlines; (ii) a matching ISO 3166 country identification grid; (iii) country area; (iv) and slope layer. Further layers include transport networks, landcover, nightlights, precipitation, travel time to major cities, and waterways. Datasets and production methodology are here described. The archive can be downloaded both from the WorldPop Dataverse Repository and the WorldPop Project website.

## Background & Summary

The global human population is projected to reach 10 billion within 40 years^1^. Continuing population growth and urbanization are projected to add 2.5 billion people to the world’s urban population by 2050, with nearly 90% of the increase concentrated in Asia and Africa^2^. The United Nations (UN) expects that continued population growth is almost inevitable until 2050, even if the current decline of global fertility accelerates. There is an 80% probability that the population of the world will be between 8.4 and 8.6 billion in 2030, between 9.4 and 10 billion in 2050 and between 10 and 12.5 billion in 2100 (ref. 3).

Detailed and contemporary spatial datasets that accurately describe human population distribution can support the measurement of the impacts of population growth, the monitoring of changes, environmental and health applications, and the planning of interventions^4^. Spatial databases of human population have found use in disease burden estimation, epidemic modelling, resource allocation, disaster management, accessibility modelling, transport and city planning, poverty mapping and environmental impact assessment amongst others^5–9^.

Recent human population mapping methodologies utilise a variety of approaches in order to assign estimated population counts to grid cells. A simple approach is to take non-spatial population data (i.e., tabular counts of population listed by administrative area) and spatially explicit administrative boundary data, and use an areal-weighting method (also known as uniform distribution or proportional allocation) to disaggregate population from census units into grid cells through the simple assumption that the population of a grid cell is an exclusive function of the land area within that pixel^10^. Gridded Population of the World (GPW) v4 (ref. 11) uses this methodology at 30 arc-seconds (approximately 1 km resolution at the equator) resolution, a dataset which details population count and density.

The areal-weighting approach does not use additional data to allocate the population within a grid cell and so has the benefit of maintaining the fidelity of input data^10^. The disadvantage of using areal-weighting as the disaggregation method is the variability of the precision of pixel-level estimates. The precision and accuracy of a given pixel is a direct function of the size of the input areal unit. Consequently, for countries where the input units are quite large, the precision of population estimates for individual pixels within that unit can be degraded^10^.

An alternative modelling approach is to use ancillary spatial data in order to improve detail, incorporating remotely sensed and other geospatial data on land cover, urban extent, accessibility, or all of these to delineate populated areas^4,5,6^. One such dataset is the Global Rural-Urban Mapping Project (GRUMP), version 1 (refs 12,13), which builds on GPW v3 (refs 14,15) to construct a common geo-referenced framework of urban and rural areas by combining census data with urban extents mapped from satellite data^16,17^.

Land cover information, including settlement extents, can be used to redistribute aggregated census counts to improve the accuracy of national scale gridded population data^18^. Gridded population distribution datasets produced using this approach generally provide more accurate results than simple areal weighting, as shown in previous studies^4,18–22^.

Recent population mapping efforts utilise a larger number of covariates, which are all related to how humans distribute themselves on the landscape, and leverage statistical relationships between these and population density data from censuses or official estimates to disaggregate areal census counts within administrative boundaries. Spatial data along with imagery analysis technologies and a multi-variable dasymetric modelling approach^23,24^ are used to produce the Landscan population datasets^25^, while a similar methodology^26,27^ is used to produce the US Census Demobase population datasets^28^. The ‘Random Forests’ regression tree-based semi-automated dasymetric modelling approach^29^ is a further example which is used to produce the WorldPop population distribution datasets^30^ (Data Citation 1, Data Citation 2). The method incorporates census data and a wide range of open access ancillary datasets in a flexible estimation technique. The combination of widely available, remotely-sensed and geospatial datasets (e.g., settlement locations, settlement extents, land cover, roads, building maps, health facility locations, satellite nightlights, vegetation, topography, refugee camps) contribute to the modelled dasymetric weights^29^. The Random Forests model is then used to generate a gridded prediction of population density at 3 arc-second spatial resolution (approximately 100 m resolution at the equator). This prediction layer is utilised as the weighting surface to perform dasymetric redistribution of census counts at country level^29^. Output suggests marked improvements in mapping accuracies over other population mapping approaches, such as areal-weighting^29,31^.

Previous population mapping work^4,20,21,23,24,26,29,32,33^ has shown that incorporating multiple spatial datasets into population mapping approaches can improve accuracy. Consequently, to support population mapping applications in the future there is a need for standardised grid definitions, standardised (contiguous) country boundaries and coastlines, and covariate layers representing different time periods that match these and that are regularly updated—all created at fine spatial resolutions. To begin to meet such needs, the WorldPop Project has produced an initial (alpha version) open access archive of 3 arc-second spatial resolution gridded datasets (hereafter referred to as 100 m tiled datasets). This paper describes the four ‘base’ standardised 100 m tiled datasets and 30 arc-second global mosaic derivations (hereafter referred to as 1 km datasets) that have been generated, as well as the production methodology used to create them. Further, the paper describes additional layers that have been incorporated into the archive, to be used to construct covariates for population modelling, and the methodologies employed.

## Methods

Base dataset creation involves processing raster topography data with vector country boundary data using a Geographical Information System (GIS) and other geospatial software. Raster images consist of a grid of pixels of particular size (spatial resolution), each pixel having a discrete (x,y) location and value. Raster images are commonly used in GIS applications, where they can represent digital elevation or terrain models of the Earth’s surface (i.e., topography). Vector datasets represent data slightly differently, utilising points (or nodes), and (in turn) lines and polygons, to represent (x,y) positions in space. Vector data can be assigned attribute information where required, and are well suited to representing boundary data.

**Figure 1:**
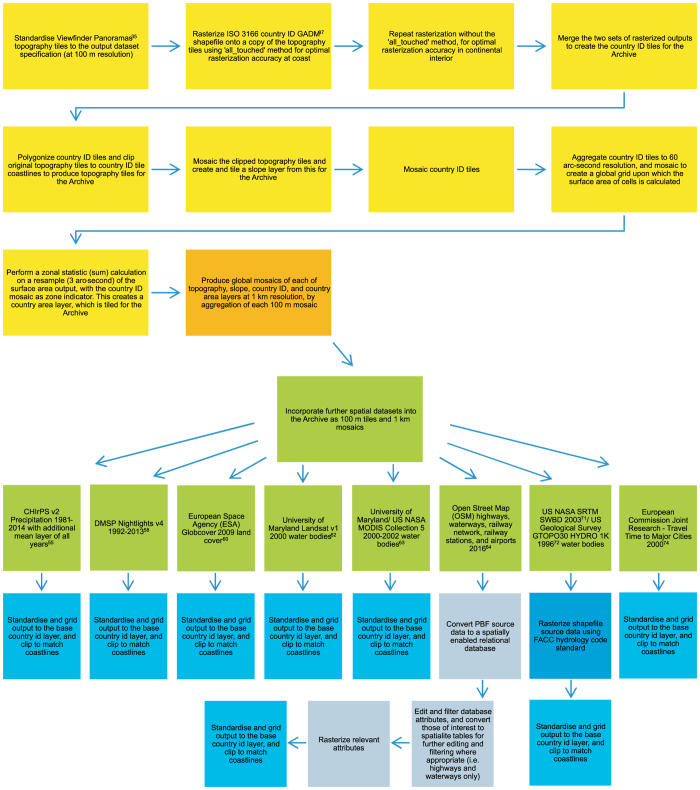
Schematic overview of the workflow used to produce the WorldPop Archive as 100 m tiles and 1 km global mosaics. The preparation of the 100 m base grids (topography, slope, country ID, and country area) is here described (yellow panels), and the methodology for preparing the accompanying 1 km mosaics defined (orange panel). Further data layers, subsequently incorporated into the archive, are summarised (in green); and the methodological approach outlined (blue). For detailed workflow and description of base datasets and further layers please see the Methods section.

Four 100 m resolution datasets form the basis of the archive outlined here: topography, standardised, gridded, and clipped to country coastal boundaries; a slope layer calculated from the topography; a country identification (ID, to the ISO 3166 standard^34^) grid; and a country area (km^2^) grid derived from this. The base topography, slope, country id, and country area grids are supplied as 100 m tiles and 1 km resolution derivatives, the latter for convenience. Input source grids for preparing base layers are summarised in Table 1, along with additional spatial data layers for incorporation into the archive. The additional layers are similarly standardised to match the grid definition and coastlines of the country ID base grid. Output base and additional layers are summarised in Table 2. See Fig. 1 for a flow chart of the workflow.

Country ID and country area grids provide essential basic metrics upon which to build population analyses. The construction of the slope layer is useful to human population studies because population densities tend to be much lower on steep slopes. Similarly, the construction of the topography layer is useful to such studies because population densities tend to be lowest at the highest elevations. Moreover, population densities are all related to landcover, infrastructure and climatic regimes, and therefore the additional layers are also potentially valuable inputs as covariates to population modelling efforts.

### Source base datasets and archive formatting

The topography data consists of the Viewfinder Panoramas dataset^35^, which is primarily US NASA Shuttle Radar Topography Mission (SRTM) data^36^ collected in the year 2000, with amendment and correction by the dataset developer, Jonathan de Ferranti^35^. The country boundary data primarily consists of the Global ADMinistrative area (GADM) v2 dataset^37^ which has been developed at the University of California^38^.

Viewfinder Panoramas data are data provided as raster tiles in hgt format. Hgt is the raw source SRTM digital elevation model (DEM) data file format^36^ and is minimally processed from the source image sensor. GADM v2 data are provided as a single ESRI shapefile. The former is at 3 arc-second horizontal resolution, whilst the effective horizontal resolution of the latter is comparable but varies according to original source administrative area and level detail.

Viewfinder Panoramas tiles are provided filled and corrected from the best available alternative sources where SRTM data are unavailable (i.e., north of 60°2'N and south of 56°S), or for some mountain and desert regions between these latitudes where there are voids and areas of phase unwrapping error^35,39^ within SRTM data. Alternative sources are usually topographic maps/ spot height elevations from maps, Landsat images, and ASTER GDEM data^35^. These sources are much more accurate than those created by simple interpolation of SRTM data, with or without the aid of SRTM30 (ref. 35). An independent review has verified the quality of the void filling^40^.

Some GADM v2 country boundaries in the continental interior are modified to GPW v4 (ref. 11) country boundaries. Similarly, some GADM v2 island and enclave country ID designations are modified to match GPW v4 and GADM v2.8 (ref. 37) data. Such modifications are to improve the general accuracy of the WorldPop country ID layer and to bring about a greater degree of standardisation with CIESIN data. A shapefile of WorldPop country boundaries, as reflected in the final raster dataset, is supplied with the archive. The forthcoming beta version of the archive will utilise new country boundaries based entirely upon CIESIN rather than GADM data.

Viewfinder Panoramas data are provided as 1,201×1,201 pixel tiles with frequent but irregular one pixel tile overlap, in geographical coordinate system (GCS) with WGS 1984 datum (EPSG:4326), and 1 m vertical resolution. These characteristics are maintained in output datasets, which otherwise utilise an unsigned integer 16 bit (for efficiency of storage) geo-tiff data format (a raster file type, with georeferencing information embedded) with 9999 nodata value and a 3 arc-second (i.e., 0.00083333333 decimal degree) cell size. Country area grids are stored as unsigned integer 32 bit due to the numerical values inherent in the data. The output topography and slope datasets exclude land area situated below sea level (a limitation of the unsigned integer format). Output datasets exclude the continent of Antarctica. These land areas typically have little population and/or seldom constitute territory belonging to developing nations and are consequently rarely of interest to those who study population. In any case, it could be considered preferable to exclude high latitudes from the dataset for reasons of error in calculated grid cell area (refer to section on Technical validation).

### Data processing software

OSGEO4W64 Geospatial Software^41^, the included Geospatial Data Abstraction Library (GDAL) v1.11.2 package^42^, ESRI ArcMap v10.3.1 and ArcInfo Workstation v9.3 GIS software^43^ are employed to produce output datasets, using a Microsoft Windows 7, 64 bit operating system (OS). OSGEO4W64 is a broad set of open source geospatial software for the Windows 64 bit environment^41^, with a command line interface that allows the user to interact with the included geospatial packages in the form of lines of text commands. The GDAL package is principally used to create the archive. GDAL is a translator library for raster and vector geospatial data formats, which presents a single raster abstract data model and vector abstract data model to the calling application for all supported formats^42^. GDAL comes with a variety of useful command line utilities for data translation and processing. Details of the various GDAL utilities employed to create the archive can be found at www.gdal.org. Although GDAL is the preferred software due to better handling of large raster datasets, employing individual ArcMap tools is preferred when functions provided by such tools are either not available or are much harder to implement in GDAL.

The processing of Open Street Map (OSM) data requires additional software and subsequent manual steps (as recommended in the documentation cited in the section ‘Incorporating further spatial datasets into Archive’). An Ubuntu Linux OS (14.04 LTS, Trusty Tahr) installation is utilised, with PostgreSQL 9.1 (ref. 44) and PostGIS 2.0 (ref. 45) database software from which spatial relational data can be exported. Osm2pgsql (ref. 46) is an OSM specific software that is used to load OSM data into databases. Subsequent database access, processing, and filtering (on the Windows platform) is provided by QGIS 2.10.1 (ref. 47) and Spatialite v4.3.0a, including the Spatalite graphical user interface (GUI) 2.0.0 (ref. 48) software. QGIS, GDAL and ArcMap software are used to extract database attributes, and to convert to raster format for subsequent tiling and mosaicking as consistent with the workflow for other datasets.

### Production of base 100 m tiled datasets

The methodology used to create the base 100 m resolution tiled global dataset is here described. The program code is implemented as a windows batch file (i.e., a plain text file which consists of a series of commands to be executed by the command line automatically when the file is run). The file is run in OSGEO4W64.

In the first instance, all (19,146) Viewfinder Panoramas topography tiles are batch converted to geo-tiff format for ease of processing in GDAL. In order to conform to the output dataset specification the nodata value is modified from −32,768 to 9,999, and the data type converted from signed integer 16 bit to unsigned integer 16 bit. Pixel values of zero (mostly water bodies) are changed to nodata using a combination of GDAL utilities (refer to lines 29–46 within the base datasets Windows batch file code that accompanies this paper). Individual tile extent is specifically defined where necessary within relevant programing loops (e.g., lines 62–84 within the base dataset code) throughout the workflow, in order to ensure that consistent base tile extents are maintained and so that end products are aligned.

In order to create country ID tiles, and clip topography tiles to the GADM coastline, three digit numerical country codes (ISO 3166 standard) are manually incorporated into the modified GADM country boundary shapefile via a join operation (using Microsoft Excel^49^ and ESRI ArcMap) and batch ‘burnt’ (written) into a copy of the topography tiles using the gdal_rasterize utility. In order that coastal boundaries of countries are most accurately represented in output data, the utility here implements an ‘all_touched’ parameter (line 81 within the code) so that the value of all pixels touched by the (GADM) polygons will be updated with the selected polygon attribute (ISO_3166), not just those pixel values that are on the line render path or whose centre point is within the polygon^50^. For any given country ID tile that is output, ID cell values will fully mask (to within one pixel) corresponding elevation cell values within the equivalent topography tile. The subsequent conversion of output country ID tiles from raster to shapefile allows all topography tiles to be clipped to the GADM coastline (using the ‘cutline’ parameter in gdalwarp) to produce the final topographic tiled product. The batch burn method is then repeated (line 125 onwards) to find accurate country borders in the continental interior. For this the 'all_touched' parameter is removed. A calculation is performed in order to combine the two batch burn outputs (coastal and continental) to produce the final country ID tiles with accurate country boundaries.

In order to create the global slope layer at 100 m resolution (and also to create other global layers down-sampled to 1 km resolution) the clipped topography tiles are mosaicked into one large image at 100 m resolution (line 220 onwards). The slope layer is created from this mosaic using the gdaldem utility, prior to assertion of the correct nodata value and data type. The slope layer is then split into tiles.

Country ID tiles are similarly mosaicked into one large image (line 266 onwards). The resulting global country ID layer later acts as an input to the ArcMap zonal statistics tool^51^, in order that the country area grid can be computed.

To create the country area grid, an ARC Macro Language (AML) script (modified from Santini *et al.*^52^) calculates the surface area of cells in a regularly spaced longitude-latitude (geographic) grid of the Earth’s surface at 60 arc-second resolution (approximately 2 km at the equator), using ESRI ArcInfo (Arc) software. 60 arc-second is the maximum resolution that can be processed (a computational limitation). AML was designed by ESRI specifically for use at the ArcInfo GIS command line. Our approach to the surface area calculation is based on the spherical approximation of the Earth’s surface (Box 1. Earth's surface area calculation) described by Santini *et al.*^52^, and is a simplification (again, for computational reasons) of the more elaborate spheroidal approximation further described and used by Santini *et al.*^52^.

Prior to the calculation of cell area using the AML script, ArcMap is used to convert country ID tiles to ESRI grid raster format and then aggregate^53^ tiles from 3 arc-second to 60 arc-second resolution. Any aggregation technique can be utilised that provides integer output. The 60 arc-second tiles are then mosaicked into a global grid. The AML script is run on the global grid. A calculation is run on the resulting cell area grid to convert cell area values within each 60 arc-second cell to that for a 3 arc-second cell size (the area of each 60 arc-second cell is divided by 400). The grid is then resampled (using ‘nearest neighbour’ method in order to maintain cell values, the default resampling method in GDAL) to 3 arc-second cell size. Subsequently, a zonal statistic (sum) calculation is performed on the resampled output (using ArcMap) with the global country ID layer as zone indicator. This creates a global output layer that expresses country area. After the adjustment of cell values from metres to kilometres and amendment of the data type and nodata value (line 281 onwards), the grid is tiled.

### Production of base 1 km global mosaics

Global mosaics of each of the topography, slope, country ID, and country area base datasets are produced at 1 km resolution (aligned with the 100 m tiled data), by aggregating each 100 m global mosaic layer using ArcMap. A 30 arc-second (i.e., 0.0083333333 decimal degree cell size) is utilised. Mean aggregation of input cells is undertaken for topography and slope layers in order to provide an accurate average downsample of all input cells that fall within each output cell. Median aggregation of input cells is undertaken for country ID and country area layers in order to most accurately choose between input cells where input datasets are binary (or where the choice of data value for each aggregated cell is overwealmingly binary, as is the case here). Median aggregation will provide the most accurate downsample available. The appropriate data types and nodata value are then asserted in the output. The slope 1 km global mosaic is derived from the float output of the gdaldem operation (with nodata values asserted), rather than from the subsequently generated integer output from which 100 m tiles are produced. This is likewise in order to provide the most accurate downsample.

### Incorporating further spatial datasets into archive

**Figure 2:**
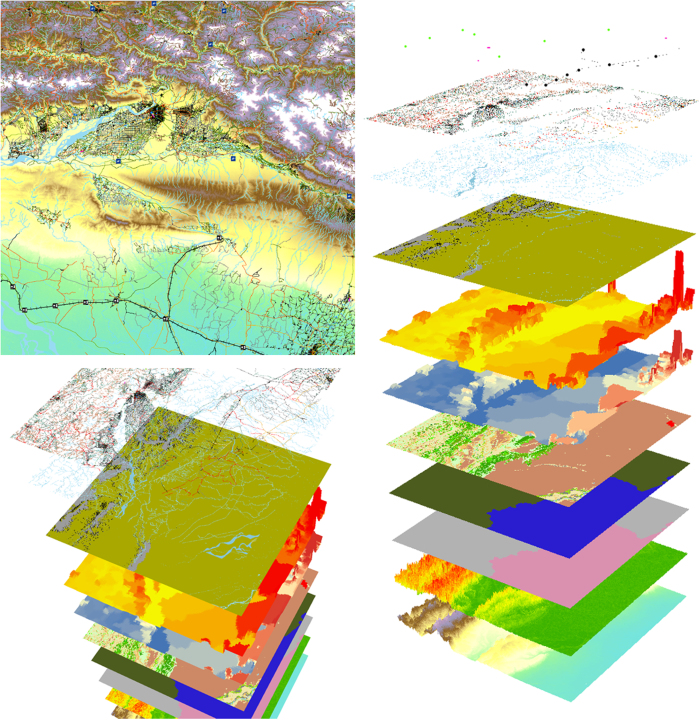
An excerpt of selected WorldPop gridded datasets at 100 m resolution, in plan view and as pseudo 3d stacks. The village of Dibyanagar, Southern Nepal, and surrounding region. Layers (in ascending order) are topography; slope; country ID; country area; Globcover^60^ land cover; GPW v4 (ref. 11) population count; GPW v4 (ref. 11) population density; Landsat^62^ water; and OSM^64^ water, highways, rail network, railway stations, runways, and heliports.

The methodology used to incorporate additional datasets into the archive is here described. The source datasets that are input are detailed in Table 1. See Fig. 2 for an example of selected outputs from the WorldPop Archive.

The ArcMap reclassify tool^54^ is used to reclassify the global country ID (1 km and 100 m) grids to values of 1 for land or 0 for sea/nodata. These reclassified grids are used to clip all further spatial datasets to WorldPop Archive coastlines.

Gridded outputs are produced using suitable resampling or aggregation techniques where applicable, as appropriate to the data type. Resampling is usually employed, and the nearest neighbour technique is applied for spatial consistency and to preserve original data values. Otherwise, aggregation techniques in ArcMap are sometimes utilised to downsample particular datasets (from 100 m to 1 km), where deemed to provide greater accuracy than the nearest neighbour resampling method. When downsampling the gridded output, nearest neighbour is best suited to categorical data where there are many values to consider. However, aggregation offers superior sampling options for some of the continuous data (e.g., topography, slope). This is also the case for categorical data that are binary, or for categorical data where the choice of data value for each aggregated cell is overwhelmingly binary (e.g., the country ID, country area, and water body data). Aggregation can provide superior output when downsampling because the method assesses all of the input cells that are encompassed by the extent of the output cell during the calculation, rather than just one cell at the centre as is the case with the nearest neighbour technique. Further, most aggregation techniques can ignore input pixel values with no data, meaning that output data are not truncated where significant zones of no data exist within an input dataset, an issue that can be particularly important (e.g., water body data, or at coastlines) when the downsample is of an order of magnitude (i.e., 100 m to 1 km).

### CHIrPS v2 Precipitation 1981–2014 with additional mean layer of all years

Climate Hazards Group Infrared Precipitation with Stations (CHIrPS) data^55^ are annual mean precipitation data provided as tiff raster layers with near global coverage. There are 35 CHIrPS layers in total (1981–2014, plus a mean layer of all years for which data are provided). Rainfall is one of the most important climatic factors affecting agricultural crop cultivation^56^. Precipitation data are useful to human population studies because population density is often spatially correlated with up to moderate amounts of rainfall^57^. Precipitation is therefore an indicator of whether climatic conditions are suitable for agriculture and settlement, and is consequently a proxy for population density.

For each layer, data are upsampled (nearest neighbour) and standardised to match the grid definition of the 1 km base country ID layer, and clipped to match the coastlines. Data are upsampled from the original spatial resolution of ~6 km. Due to the numerical values inherent in the data, a nodata value of 65535 is utilised rather than the standard 9999 value that would otherwise be preferred. A nodata value of 65535 falls outside of the range of values in the dataset. Layers are subsequently resampled (nearest neighbour) to match the grid definition of the 100 m base country ID layer, and again clipped to match coastlines, before each layer is tiled to base tile extents. CHIrPS data are only available between latitudes 50 degrees North and 50 degrees South. Consequently all tiles created outside of these latitudes are redundant and so removed. GDAL is used throughout the process.

### DMSP nightlights v4 1992–2013

DMSP-OLS Nighttime Lights v4 Time Series data^58^ are annual night light intensity data provided as tiff raster layers with near global coverage. There are 66 Nightlights ~1 km spatial resolution layers in total (1992–2013, 22 Average Visible, 22 Stable Lights, and 22 Cloud Free Coverages). Night lights data are useful to human population studies because night light intensity is a strong indicator of degree of urbanisation, and consequently a proxy for population density^59^.

For each layer, data are first standardised to match the grid definition of the 1 km base, and then clipped to match the coastlines. Each raster is subsequently resampled (nearest neighbour) and standardised to match the grid definition of the 100 m base and once more clipped, this time to match the coastlines of the 100 m base. Output is tiled to base grid extents. Nightlights data are only available between latitudes 75 degrees North and 65 degrees South. Consequently all tiles created outside of these latitudes are redundant and so removed. GDAL is used throughout the process.

### European space agency (ESA) globcover 2009 land cover

Globcover 2009 (ref. 60) is a global land cover dataset provided as a tiff raster layer. Land cover data are useful to human population studies because land cover classification often categorises urban and agricultural areas, a useful proxy for population density^18^.

The Globcover 2009 layer is first upsampled (nearest neighbour) from the original spatial resolution of 250 m and standardised to match the grid definition of the 100 m base. The data are clipped to match 100 m base coastlines and subsequently tiled to base tile extents. GDAL is used throughout the process. The 100 m layer is resampled^61^ (nearest neighbour) to 1 km resolution using ArcMap.

### University of Maryland Landsat v1 2000 water bodies

Landsat v1 water body data^62^ are provided as 30 m spatial resolution tiff raster tiles, defining lakes and rivers, with global coverage. Inland water body data are useful as a basic metric upon which to build human population analyses (being particularly valuable for use in masking out areas of non-human habitation); and can be a useful indicator of possible locations of high population density, especially for dry climatic regions, where supported by other datasets.

The nodata value of the tiles are asserted in GDAL before ArcMap is used to create a mosaic dataset, with a new geographical coordinate system (GCS) projection with WGS 1984 datum (EPSG:4326), to which the Landsat tiles are added (resampled using nearest neighbour technique in order to maintain categorical values). The mosaic is then exported to a new raster. Using GDAL the new raster is downsampled (nearest neighbour) and standardised to match the grid definition of the 100 m base layer, and then clipped to match the coastlines of the 100 m base. This 100 m global grid is tiled to base grid extents using GDAL, and also aggregated (using median method) to 1 km resolution using ArcMap before the nodata value is asserted.

### University of Maryland/US NASA MODIS collection 5 2000–2002 water bodies

MODIS water body data^63^ (lakes and rivers) are provided as 250 m spatial resolution tiff raster tiles with global coverage, and are similarly useful as a basic metric upon which to build population analyses. These data are a potential indicator of high population density where supported by other datasets.

The MODIS tiles are mosaicked into a global grid and upsampled (nearest neighbour) and standardised to match the grid definition of the 100 m base. The 100 m global raster is clipped to match the coastlines of the 100 m base, and then tiled to base grid extents. GDAL is used throughout the process. The global raster is then aggregated (using median method) to 1 km resolution, using ArcMap, prior to the nodata value being asserted.

### Open street map (OSM) waterways, highways, railway network, railway stations, and airports 2016

OSM is global mapping data provided as a database^64^. OSM data have an effective resolution typically comparable with SRTM1 at ~30 m but varies according to original source data. OSM highway, railway network, railway stations, and airport mapping data are useful to human population studies in providing strong indicators of degree of urbanisation, and consequently are proxies for population density^29,31^. For example regions with many road and rail intersections are likely to be locations of higher population, as are regions with high road and rail density. Railway stations and airports are similarly likely to be proximal to areas of high population. OSM waterway data are useful as a basic metric upon which to build population analyses; and include urban waterways such as drains, ditches and sewers, as well as natural waterways such as lakes and rivers. As such, OSM waterway data can improve the precision and accuracy of population datasets^65^.

OSM data use a system of nodes, ways, and relations to define points in space, linear features/area boundaries, and the way in which these attributes work together, respectively. Tags are used to categorise and label each attribute. The frequency of contributions by individual users will refine source data^66^, as often will contributions from professional cartographic organisations. Although effective resolution of OSM data is excellent, the lack of sufficiently standardised user tagging of attributes can cause inaccuracies and difficulties in map rendition.

The workflow for integrating OSM data into the archive is more elaborate than is the case for other datasets because of the source database Protocolbuffer Binary Format (PBF)^67^. PBF is intended as an alternative to the XML format, both of which are used to store OSM ‘planet’ (i.e., global) data, which is updated weekly. PBF is about half of the size of a gzipped (XML) planet and about 30% smaller than a bzipped (XML) planet, and is about 5×faster to write than a gzipped planet and 6×faster to read than a gzipped planet. The format was designed to support future extensibility and flexibility^67^.

An Ubuntu Linux OS with PostgreSQL 9.1 and PostGIS 2.0 installed is used at command line to download and convert the PBF file into a spatially enabled relational database, from which data can subsequently be exported and converted into raster format for inclusion in the WorldPop Archive. Osm2pgsql is used to load the OSM data into the Postgres database^68^. The methodology used is documented at https://switch2osm.org/loading-osm-data/.

Each table (point, line, polygon, and (major) road) within the resulting database is accessed using QGIS software. The QGIS Database Manager SQL window is utilised to add a unique identifier (column) to each attribute (row) within each table, and to make this the primary identifying field (refer to lines 637–643 within the further datasets Windows batch file code that accompanies this paper). The ‘way’ type of the polygon table is also defined as ‘multipolygon’ in order to allow further processing. The database attributes of interest are then converted to spatialite tables. Converting to spatialite format^69^ tends to allow greater and faster manipulation of spatial data. This is especially useful for highways (see section OSM highways) where Spatialite software is utilised to further edit tables. Attribute extraction from the database, and conversion to raster format can then take place, using a combination of QGIS, GDAL and ArcMap.

Ultimately, for each OSM sub-dataset (i.e., waterways, highway, etc.), source data are sampled (nearest neighbour) to 100 m and standardised to match the grid definition of the 100 m base. Data are tiled and coastlines clipped to match 100 m base coastlines. The 100 m tiles are mosaicked and then aggregated (maximum) to 1 km resolution using ArcMap. The use of the maximum aggregation technique ensures that input data values, or prioritised input data values, are accurately transferred to downsampled grids. Further specific workflow for each OSM sub-dataset are here discussed.

*OSM waterways*. In the first instance three types of water attributes are extracted from the postgres database by filtering using the Query Builder window in QGIS. These attributes are general ‘waterway’ polylines (streams, rivers, drains, ditches, etc.), riverbank polygons (where rivers, or similar, have quantifiable width at source data resolution), and lake polygons (or similar). The filtered attributes are converted to three spatialite tables. For general ‘waterway’ tagged polylines the filter used is ‘waterway’!=’NULL’. For relevant 'waterway' and 'natural' polygons the filter is ‘waterway’=‘riverbank’, ‘natural’=‘lake’, and ‘natural’=‘wetlands’. Other variants and/or misspellings of these tags are also included in the filter for completeness where applicable.

The spatialite tables for waterways and lakes are converted to global rasters (in tiff format) at 100 m resolution using gdalwarp. Riverbanks are converted from spatialite to shapefile format using QGIS and rasterized using ArcMap (using the maximum area method in order that the single feature with the largest area within the cell yields the attribute to assign to the cell^70^). The three rasters are reclassified (using the reclassify tool in ArcMap) so that any 0 values are redesignated as 1. This is necessary as some attributes are defined as 0 in the output. A uniform designation of 1 for all water features is required for simplicity. Nodata values are asserted for each raster, which are then tiled (using GDAL) to base grid tile extents for ease of subsequent processing.

Each of the three tiled datasets are mosaicked together (lines 782–809 within the further datasets code), using gdalwarp, so that lake tiles are superimposed onto riverbank tiles, which are in turn superimposed onto waterway tiles—to form a single tiled dataset. Coastlines in this tiled output are clipped to the coastlines in the country ID tiled dataset. A calculation is performed to amend for where some high latitude tiles have zero values assigned for land, where a nodata value is required. The tiles are mosaicked and aggregated (using maximum method) to 1 km resolution using ArcMap. The nodata value is then asserted.

In the archive, a pixel value of 1 is usually used to denote the presence of an attribute feature in OSM data where the data are not complex. All 'waterway' tagged features (streams, rivers, drains, etc.) in the osm data are denoted by this value. Relevant 'waterway' and ‘natural’ tagged features (lakes, wetlands, riverbanks) are similarly denoted.

*OSM highways*. Following a similar method as for OSM waterways, the highway polyline attribute is extracted from the database in QGIS by filtering (using ‘highway’!=’NULL’), and converted to a spatialite table. The spatialite table is then edited in Spatialite GUI, using the SQL statement window, in order to create a ‘Priority’ field with which to rank the standard of roads in the highway network. Priority values are later preserved as pixel values when tables are converted to raster format, with higher priority roads taking precedence in the subsequent raster mosaicking process. Highway tags are assigned a priority value in the spatialite table. Priority value assignment is detailed in Supplementary Table 1. Assignment simplifies highway tagging to make it manageable for display in raster format.

The ‘bridge’ and ‘tunnel’ polyline attributes (henceforth referred to as ‘links’) are together extracted from the database by filtering (with certain exceptions detailed in the code), and converted to a spatialite table. OSM highways and rail networks over/under water (e.g., bridges and tunnels at estuaries, narrow sea ways, etc.) are unfortunately removed during the later clipping of highway and rail network rasters to base coastlines. The addition of an OSM links attribute to the archive restores those transport links to road and rail networks in the dataset. Consequently road and rail network data can be clipped to coastlines and yet remain contiguous.

As is the case for highways, the links spatialite table is edited in Spatialite to create a ‘Priority’ field. To this field links are given an arbitrary priority value to differentiate them from the rest of the road (and rail) network. Links are assigned the arbitrary priority of 30 and are ultimately placed in the archive OSM highway dataset.

The links spatialite table is converted to a global raster at 100 m resolution, using GDAL. The highways spatialite table is further filtered into separate spatialite tables for each highway priority, and then each table is converted to a global raster at 100 m resolution, using the polyline to raster tool in ArcMap. A separate table for each highway priority is necessary due to memory limitations in ArcMap. Output highway rasters are then mosaicked (using maximum method) into a single raster (using ArcMap) and the nodata value asserted using GDAL.

The single highway mosaic (lines 975–1000) and the links raster (lines 1032–1057) are each tiled to base grid extents and the country ID shapefile tiles (created as part of processing to construct base layers) used to clip the highway tiles to base coastlines (lines 1002–1030), using GDAL. In order to generate a finished links raster that is clipped to the area of interest (i.e., over/under water) beyond coastlines, a mask of land area is created from country ID tiles via a series of calculations in GDAL. Links that occur on land are eliminated, leaving only those that occur beyond coastlines. The output links tiles are then merged with the highway tiles that have been clipped to coastlines. Output is mosaicked at 100 m resolution using GDAL, then aggregated (using maximum method) to 1 km resolution using ArcMap, and nodata values asserted.

*OSM rail network*. Similarly, railway polyline attributes are extracted from the database, filtered in QGIS to obtain the desired rail (network) attributes, and then converted to a spatialite table. The filter used is detailed in the code, and identifies the many tags that constitute the main rail network, including variants/ misspellings, whilst disregarding other rail related features that have no relevance.

The spatialite table is subsequently converted to a global raster at 100 m resolution using GDAL, and the ArcMap reclassify tool utilised to reclassify 0 values to 1. The nodata value is asserted, and the raster tiled to base grid extents (lines 1236–1261) using GDAL. Country ID shapefile tiles (created as part of processing to construct base layers) are used to clip rail network tiles to base coastlines. Output is then mosaicked, aggregated (using maximum method) to 1 km resolution using ArcMap, and nodata values asserted.

*OSM rail stations & airports (heliports and runways).* Railway point, and aeroway point and polyline attributes are each separately extracted from the database, filtered in QGIS to obtain railway station (including underground stations), heliport, and airport runway attributes, then each converted to shapefiles. The filters used are ‘railway’=’station’, ‘railway’=’subway_station’, ‘aeroway’=’heliport’ and ‘aeroway’=’runway’. Variants of these tags are also included in the filter for completeness where applicable.

Each shapefile is converted to a global raster at 100 m resolution and 0 values reclassified to nodata, using ArcMap. Nodata values are asserted for each raster, and each is then tiled to base grid extents (lines 1338–1363, lines 1388–1413, and lines 1437–1462, for each of stations, heliports, and runways respectively) using GDAL. Global rasters are aggregated (using maximum method) to 1 km using ArcMap, and nodata values asserted. Finally, very high latitude tiles for each of stations, heliports, and runways are deleted where they contain only 0 nodata values, as these tiles are redundant.

Tags for railway underground stations are inconsistently used in OSM source data. Some underground stations use the regular (overground) 'station' tag (denoted by the pixel value of 1 in the archive). Where underground stations are tagged using the 'subway' tag this is denoted by a pixel value of 2 in the archive.

Aviation heliports in the OSM source data are sometimes differentiated from helipads in tagging. The latter are denoted by a pixel value of 2 in the archive.

### US NASA SRTM SWBD 2003/US geological survey GTOPO30 HYDRO 1 K 1996 water bodies

SRTM (SWBD) and GTOPO30 Hydro 1 K water body data^71,72^ (lakes and rivers) are provided as shapefile tiles from data sources of ~30 m to ~1 km resolution, with global coverage. Inland water body data are useful as a basic metric upon which to build population analyses (e.g., masking out areas of non-human habitation), and as a potential indicator of high population density where supported by other datasets.

For each shapefile tile, an ‘FC’ field is added to the attribute table and populated with the numerical part of the FACC Hydrology (BH000, Inland water; BA040, Water (except inland); BH080, Lake/pond; and BH140, River/Stream) code standard^73^. Each shapefile is rasterized (using the FC attribute field to identify the value to burn into the raster) and standardised to match the grid definition of the 100 m archive base layer. The rasters are mosaicked into a global grid and then clipped to match the coastlines of the 100 m base. The clipped mosaic is then tiled to base grid extents using GDAL, and aggregated (using median method) to 1 km resolution (using ArcMap) before the nodata value is asserted.

### European commission joint research—travel time to major cities 2000

The Travel Time to Major Cities data^74^ are provided as a 1 km spatial resolution flt (ArcInfo 32 bit binary float grid format) raster layer, with global coverage. The layer utilised here depicts the travel time to settlements with population greater than 50,000 people. Such data are useful to human population studies because travel time is primarily a function of distance. Therefore travel time to major cities is a strong indicator of degree of urbanisation, and consequently represents a proxy for population density. Population density tends to be lowest in the most remote areas with longest travel times, whereas locations with short travel times are likely to be more urbanised and so be locations of higher density.

The layer is first standardised to match the grid definition of the 1 km base. Due to the numerical values inherent in the data, a nodata value of 65535 is utilised rather than the standard 9999 value that would otherwise be preferred. The raster is clipped to match 1 km base coastlines and then resampled (nearest neighbour) to match the grid definition of the 100 m base. Output is once again clipped, this time to match the coastlines of the 100 m base, and the raster tiled to base grid extents. GDAL is used throughout the process.

### Code availability

The program code to produce the WorldPop Archive 100 m tiled (and 1 km global mosaic) base datasets, the code to incorporate further spatial datasets into the archive, as well as the AML script used in the production of the country area base layer are available for download^75^.

The code consists of two windows batch files containing mainly GDAL commands for use at command line in OSGEO4W 64 shell, and an AML script for use with ESRI ArcInfo Workstation software. Each file is internally documented in order to explain purpose (including a description of the GIS-specific spatial operations that the file performs) and, when required, to guide the user in file customization. File internal documentation should be consulted in combination with this paper.

## Data Records

All output datasets described in this article (Data Citation 3, Data Citation 4, Data Citation 5, Data Citation 6, Data Citation 7, Data Citation 8, Data Citation 9, Data Citation 10, Data Citation 11, Data Citation 12, Data Citation 13, Data Citation 14, Data Citation 15) are publicly and freely available both through the WorldPop Dataverse Repository (https://dataverse.harvard.edu/dataverse/WorldPop) and the WorldPop website (www.worldpop.org.uk/data/lloyd_hires_global_data_paper). The datasets stored in the WorldPop Dataverse Repository represent the datasets produced at the time of writing, and will be preserved in their published form. The datasets stored on the WorldPop website may be updated, particularly the OSM data which will be expanded and updated annually. Datasets of interests can be obtained by downloading the corresponding zipped archive files (Table 3).

## Technical Validation

Datasets produced by the WorldPop Project for this paper have been obtained by simply processing input source data to produce consistent 100 m and 1 km outputs. The source data are already validated by other independent studies (e.g., Hormann^40^, Rabus *et al.*^76^, Rodriguez *et al.*^77^, Brigham *et al.*^78^, Center for International Earth Science Information Network (CIESIN)—Columbia University^79^, Funk *et al.*^55^, Henderson *et al.*^80^, Min *et al.*^81^, European Space Agency (ESA) and Université Catholique De Louvain (UCL)^60^, Feng *et al.*^82^, Carroll *et al.*^83^, Iwao *et al.*^84^, and Varga and Bašić^85^). Open Street Map data does not comply with standard data quality assurance procedures^86^ because OSM is ‘volunteered geographical information’, provided by any number of individual contributors. However, OSM data have intrinsic quality assurance through analysis of the number of contributions for a given spatial unit. The assumption that as the number of contributors increase then so does the quality of the data is known as ‘Linus’ Law’. Recent studies show that for OSM data this rule applies with regard to positional accuracy^86^. Consequently OSM data quality is comparable to the most accurate datasets utilised in this paper—with the caveat that map coverage must be considered satisfactory for any given spatial unit under scrutiny^87^.

### Cell surface area calculation: grid resolution induced error

To produce the WorldPop country area base grid, the surface area of cells in a regularly spaced longitude-latitude (geographic) grid of the Earth’s surface are calculated. Our approach to the surface area calculation is based on the spherical approximation of the Earth’s surface (described by Santini *et al.*^52^) and uses a simplified approximation of the Earth spheroid with grid cells of 60 arc-second resolution (i.e., 0.016666666 decimal degree). In order to provide a validation of the approximation we here assess grid resolution induced cell area error by comparison of calculated area values of the 60 arc-second resolution grid cells with those summed for the corresponding 3 arc-second resolution (i.e., 0.0083333333 decimal degree) grid cells.

The areas of twenty consecutive cells at three different latitudes (low, medium, and high) are calculated at the finer resolution, ascending in latitude within a grid tile. The area of a 20×20 cluster of the same cells (i.e., 400 cells) is then determined at each latitude. This provides coverage equivalent to a single 60 arc-second cell at each latitude. It follows that the difference in area values between the 3 arc-second and 60 arc-second resolution grids can be assessed for different latitudes, and error considered. Our analysis (Supplementary Table 2a–c) demonstrates that mean absolute error (MAE) between the two grids is trivial from low (0.0°, 0 m^2^ MAE) to middle latitudes (51.0°, 0.49 m^2^ MAE). This is because cell area is relatively large (of the order of several thousand m^2^) at such latitudes. Error only becomes significant at very high latitudes (89.9°, 0.62 m^2^ MAE) where cell area is extremely small (of the order of a single m^2^ or less). The effect of this error at very high latitudes will be especially acute for larger countries.

The issue of grid resolution induced error in the cal3culation of cell area is therefore of little concern to our study. Our grid approximation is acceptable in the context of human population studies because human population is negligible at very high latitudes.

## Usage Notes

It is hoped that this alpha version of the WorldPop Archive will assist researchers by providing a uniform base upon which analysis of population distributions can be performed. Such analysis will in turn allow measurement of the impacts of population growth, the monitoring of changes, environmental and health applications, and the planning of interventions^4^.

The archive provides resampled, co-registered, and ready to use spatial data layers at two different resolutions that users can employ for modelling and analysis purposes. The archive can be edited to fit user requirements with the minimum of effort.

The WorldPop Archive described in this paper is not a comprehensive set of layers for population mapping applications, but rather it represents an initial effort to assemble and provide the research community with a set of globally consistent, open-access, and harmonized layers. The archive has limitations mostly associated with input source data spatial coverage and resolution. This may limit the usefulness of the WorldPop gridded output to the end user (e.g., CHIrPS and DMSP Nightlights), as might the acquisition period of particular data. For many of the datasets within the archive there is a substantial lag of 10–15 or more years from acquisition date to present. Consequently in many countries/areas substantial local changes, especially due to human activities or mobility, are likely to have occurred and so data may be substantially outdated in these instances. Thus, if needed, users are encouraged to complement these layers with more recent and better global or country specific data including geolocated mobile information from call data records^33,88^ and social media^89^.

We intend to add further datasets to the archive in the future to improve spatial and temporal coverage, as well as to broaden the type of data within the archive. Datasets such as the Global Human Settlement Layer (GHSL)^90^; ESA CCI global land cover v1.6.1, water bodies v4 (ref. 91); and VIIRS nighttime lights^92^ will be incorporated into the archive soon. The archive is part of an ongoing project, with revisions and many more spatial layers to be released in the future.

Limitations of the archive base gridding process include the potential for small islands to be absent from the country ID base grid because the islands are not present in source GADM v2 or GPW v4 data. This has the consequence that corresponding small island topographic or other spatial data are excluded from the WorldPop gridded product. Further, where coastlines differ between country ID and input topography/other spatial layer, coastal pixels (with a data value) may be removed from the output grid during standardisation (clipping) to the country ID layer. Similarly, pixels (with no data value) adjacent to coastlines in input topography/other spatial layer will remain in the output grid where they occur within the coastal boundary of the country ID layer—i.e., there is no data interpolation at coastal boundaries.

The Travel Time to Major Cities layer is based on a complex and heavy modelling combination of multiple datasets^74^, some of which are included in the WorldPop Archive. The end user should be aware that consequently this layer is not independent of these datasets and thus, in order to avoid endogeneity, it should not be used in population studies along with them.

## Additional Information

**How to cite this article:** Lloyd, C. T. *et al.* High resolution global gridded data for use in population studies. *Sci. Data* 4:170001 doi: 10.1038/sdata.2017.1 (2017).

**Publisher’s note:** Springer Nature remains neutral with regard to jurisdictional claims in published maps and institutional affiliations.

## Supplementary Material



Supplementary Information

## Figures and Tables

**Table 1:** Input datasets, used to produce the WorldPop Archive 100 m tiles and 1 km global mosaics.

**Name**	**Acquisition Year**	**Source**	**Version, Publication Year**	**Data Type**	**Spatial Resolution**	**Format/ Pixel Type & Depth**	**Spatial Reference**	**Spatial Coverage**
Viewfinder Panoramas	~2000	de Ferranti, J^35^	26/05/14	Elevation, continuous raster	Typically 3′′ (~90 m)	HGT tiles/ int16	GCS WGS 1984	Global
GADM	2012/ 2015	Global ADMinistrative Areas (GADM)^37^	v2/v2.8	Global Admin. Boundaries (Country borders), categorical vector	Comparable to 3′′ (~90 m)	ESRI polygon shapefile	GCS WGS 1984	Global
Gridded Population of the World (GPW)	2010	Center for International Earth Science Information Network (CIESIN), Columbia University^11^	v4, 2014	Global Population Count/ Density (Country borders), continuous raster	30′′ (~900 m)	Geo-tiff/ flt32	GCS WGS 1984	Global
Climate Hazards Group Infrared Precipitation with Stations (CHIrPS)	1981–2014	Funk, C., *et al.*^55^	v2, 2015	Annual precipitation, continuous raster	~180′′ (~6 km)	Geo-tiff/ flt32	GCS WGS 1984	Between latitudes 50° North and 50° South
DMSP Nightlights Time Series	1992–2013	US NOAA National Geophysical Data Center^58^	v4, 2014	Night lights intensity, continuous raster	30′′ (~900 m)	Geo-tiff/ uint8	GCS WGS 1984	Between latitudes 75° North and 65° South
Globcover	2009	European Space Agency (ESA) & Université Catholique De Louvain (UCL)^60^	2010	Land cover, categorical raster	7.5′′ (~250 m)	Geo-tiff/ uint8	GCS WGS 1984	Global
Landsat	<2000	University of Maryland, Department of Geography^62^	v1, 2015	Inland water bodies, categorical raster	1′′ (~30 m)	Geo-tiff tiles/ uint16	UTM WGS 1984	Global
MODIS MOD44W Collection 5	2000–2002	University of Maryland, Department of Geography/ US NASA^63^	2009	Inland water bodies, categorical raster	7.5′′ (~250 m)	Geo-tiff tiles/ uint8	GCS WGS 1984	Global
Open Street Map (OSM)	2016	OpenStreetMap Foundation (OSMF) & Contributors^64^	15/01/16	General mapping, categorical vector	Comparable to 1′′ (~30 m)	PBF database	GCS WGS 1984	Global
SRTM SWBD	~2000	US NASA & US National Geospatial-Intelligence Agency (NGA)^71^	v2, 12/03/03	Inland water bodies, categorical vector	Comparable to 1′′ (~30 m)	ESRI polygon shapefile tiles	GCS WGS 1984	Between latitudes 60°2' North and 56° South
GTOPO30 HYDRO 1 K	<1996	US Geological Survey EROS Data Center^72^	1996	Inland water bodies, categorical vector	Comparable to 30′′ (~900 m)	ESRI polygon shapefile tiles	GCS WGS 1984	Greater than latitudes 60°2' North and 56° South
Travel Time To Major Cities	2000	Nelson, A. (European Commission Joint Research Centre Global Environment Monitoring Unit)^74^	2008	Travel time, continuous raster	30′′ (~900 m)	Flt/flt32	GCS WGS 1984	Global
Input datasets are here described. Data source, version, format, and spatial and temporal statistics are summarised. The table shows input grids from which WorldPop base grids (topography, slope, country ID, and country area) are prepared, and shows additional datasets subsequently standardised and gridded for inclusion in the archive. Refer to the Methods section for a more detailed description of how base datasets and the additional datasets are produced.								

**Table 2:** Output WorldPop Archive datasets (100 m tiles and 1 km global mosaics).

**Name**	**Acquisition Year**	**Source**	**Version, Publication Year**	**Data Type**	**Spatial Resolution**	**Format/ Pixel Type & Depth**	**Spatial Reference**	**Spatial Coverage**
Topography	~2000	de Ferranti, J^35^	26/05/14	Elevation, continuous raster	3′′ (~90 m)	Geo-tiff/ uint16	GCS WGS 1984	Global
Slope	Derived from topography	Slope, continuous raster	3′′ (~90 m)	Geo-tiff/ uint16	GCS WGS 1984	Global		
Country ID	2012,2015/2010	Global ADMinistrative Areas (GADM)^37^/ Center for International Earth Science Information Network (CIESIN), Columbia University^11^	v2,v2.8/v4, 2014	Country borders, categorical raster	3′′ (~90 m)	Geo-tiff/ uint16	GCS WGS 1984	Global
Country area	Derived from calculated Earth surface area grid and the country ID layer	Country area, categorical raster	3′′ (~90 m)	Geo-tiff/ uint32	GCS WGS 1984	Global		
Climate Hazards Group Infrared Precipitation with Stations (CHIrPS)	1981–2014	Funk, C., *et al.*^55^	v2, 2015	Annual precipitation, continuous raster	3′′ (~90 m)	Geo-tiff/ uint16	GCS WGS 1984	Between latitudes 50° North and 50° South
DMSP Nightlights Time Series	1992–2013	US NOAA National Geophysical Data Center^58^	v4, 2014	Night lights intensity, continuous raster	3′′ (~90 m)	Geo-tiff/ uint16	GCS WGS 1984	Between latitudes 75° North and 65° South
Globcover	2009	European Space Agency (ESA) & Université Catholique De Louvain (UCL)^60^	2010	Land cover, categorical raster	3′′ (~90 m)	Geo-tiff/ uint16	GCS WGS 1984	Global
Landsat	<2000	University of Maryland, Department of Geography^62^	v1, 2015	Inland water bodies, categorical raster	3′′ (~90 m)	Geo-tiff/ uint16	GCS WGS 1984	Global
MODIS MOD44W Collection 5	2000–2002	University of Maryland, Department of Geography/ US NASA^63^	2009	Inland water bodies, categorical raster	3′′ (~90 m)	Geo-tiff/ uint16	GCS WGS 1984	Global
Open Street Map (OSM)	2016	OpenStreetMap Foundation (OSMF) & Contributors^64^	15/01/16	Highways, waterways, rail network, rail stations, airports, categorical raster	3′′ (~90 m)	Geo-tiff/ uint16	GCS WGS 1984	Global
SRTM SWBD/GTOPO30 HYDRO 1 K	~2000/ <1996	US NASA & US National Geospatial-Intelligence Agency (NGA)^71^/US Geological Survey EROS Data Center^72^	v2, 12/03/03 /1996	Inland water bodies, categorical raster	3′′ (~90 m)	Geo-tiff/ uint16	GCS WGS 1984	SRTM between latitudes 60°2' North and 56° South/GTOPO at greater than latitudes 60°2' North and 56° South
Travel Time To Major Cities	2000	Nelson, A. (European Commission Joint Research Centre Global Environment Monitoring Unit)^74^	2008	Travel time, continuous raster	3′′ (~90 m)	Geo-tiff/ uint16	GCS WGS 1984	Global
Output base datasets (topography, slope, country ID, and country area) and additional datasets are here described. Data source, version, format, and spatial and temporal statistics are summarised.								

**Table 3:** Name, description, and DOI of the high resolution global gridded datasets described in this paper.

**Name**	**Description**	**Dataverse DOI**
100 m base topography (tiled)	SRTM-based elevation (m)	10.7910/DVN/ET52ON
100 m base slope (tiled)	SRTM-derived slope (degree)	10.7910/DVN/VKAYE8
100 m base country code ID (tiled)	Numeric ISO-3166 country code IDs	10.7910/DVN/BAOZPR
100 m base country area (tiled)	Country area (km^2^)	10.7910/DVN/UBJ3WQ
100 m CHIrPS v2 (tiled)	Precipitation (mm/yr)	10.7910/DVN/89TAOX
100 m nightlights v4 (tiled)	DMSP nightlights (average of visible band digital number values)	10.7910/DVN/VO0UNV
100 m Globcover 2009 (tiled)	MERIS-based landcover	10.7910/DVN/XALRAG
100 m Landsat inland water 2000 (tiled)	Landsat-based waterbodies	10.7910/DVN/JYJINK
100 m MODIS global water (tiled)	MODIS-based waterbodies	10.7910/DVN/XSGAG3
100 m OpenStreetMap (tiled)	Waterways, highways, railway network, railway stations, airports	10.7910/DVN/VEO2BQ
100 m SRTM SWBD (tiled)	SRTM-based waterbodies	10.7910/DVN/G6X1ZS
100 m Travel Time To Major Cities 50 K (tiled)	Accessibility to settlements with more than 50,000 inhabitants	10.7910/DVN/K8HYXZ
1 km global mosaics	All above datasets resampled to 1 km resolution	10.7910/DVN/ADYEZK

## Earth's surface area calculation.

As elucidated by Santini *et al.*^52^, the surface area S of a generic cell of a (global) grid, comprised between λ1 and λ2 degrees of longitude and between φ1 and φ2 degrees of (authalic) latitude^93^ can be calculated^94^ as:

(1)S=∫φ1φ2R(∫λ1λ2Rcosφdλ)dφ=∫φ1φ2R2cosφdφ∫λ1λ2dλ=R2(λ2−λ1)∫φ1φ2cosφdφ

giving:

(2)S=R2(λ2−λ1)(sinφ2−sinφ1)

Where

0≤λ1≤λ≤λ2≤2π

−π/2≤φ1≤φ≤φ2≤π/2

R=the (authalic) radius of the sphere representing the Earth.

φ1, φ2 and λ1, λ2 are generic coordinates of a point belonging to the cell, expressed in radians (π radians=180 degrees).
