# Supplementary material for: High resolution global gridded data for use in population studies
Source: Sci Data. 2017 Jan 31;4:170001. doi: 10.1038/sdata.2017.1 (PMC5283062; doi:10.1038/sdata.2017.1)
Supplement: Supplementary Information [file sdata20171-s2.doc]

**Supplementary Tables**

**Table of Contents**

Supplementary Table 1…………………………………………………………………………………………………………….…………………………………………………………………………………………...…2

Supplementary Table 2a.…………………………………………………………………………………………………….……………………………………………………………………………………….….…….…3

Supplementary Table 2b………………….………..……………………………………………………………………………………………………………………………………….………………………..…….……4

Supplementary Table 2c…………………………………………………………………………………………………………………………………………….……………………………………………………….……5

| **Priority Value** | **Highway Tag** |
| --- | --- |
| 1 | 'proposed' OR 'construction' |
| 2 | 'path' OR 'footway' |
| 3 | 'track' OR 'bridleway' OR 'disused' OR 'unsurfaced' OR 'abandoned' OR 'trail' OR 'byway' OR 'unknown' OR 'unmarked_route' |
| 4 | 'service' OR 'services' |
| 5 | 'living_street' OR 'pedestrian' |
| 6 | 'unclassified' OR 'road' OR 'yes' |
| 7 | 'residential' |
| 8 | 'tertiary_link' |
| 9 | 'tertiary' |
| 10 | 'secondary_link' |
| 11 | 'secondary' |
| 12 | 'primary_link' |
| 13 | 'primary' |
| 14 | 'trunk_link' |
| 15 | 'trunk' |
| 16 | 'motorway_link' |
| 17 | 'motorway' |

**Supplementary Table 1. Priority values for OSM highway tags in the spatialite table.**

**OSM highway tags are assigned a priority value in the spatialite spatial relational table in order to simplify the complex tagging of highway data in the OSM database. Assignment of priority values makes highway tagging more manageable for display in raster format. Variants and/or misspellings of highway tags are included in assignment of priority values for completeness where applicable, but are not included in the table for reasons of brevity.**

| **3 arc-second resolution** | | | | | | | | | | | | | | | |
| --- | --- | --- | --- | --- | --- | --- | --- | --- | --- | --- | --- | --- | --- | --- | --- |
| Px no. from the tile bottom | R2 (m) | CellSizeDeg | CellSizeRad | LowerLat | UpperLat | LowerLatRad | UpperLatRad | SinLowLatRad | SinUppLatRad | SinUpp-SinLow | Px AREA (m2) 3as resolution | Px AREA (m2) 60as resolution | Error | ABS (Error) | **MAE** |
| 1 | 6371009 | 0.0008333333 | 0.000014544409851509 | 0.0000000000 | 0.0008333333 | 0 | 1.45444E-05 | 0.000000000000000000 | 0.000014544409851 | 0.000014544409850997 | 8586.35 | 8586.35 | 0.00 | 0.00 | **0.00** |
| 2 | 6371009 | 0.0008333333 | 0.000014544409851509 | 0.0008333333 | 0.0016666666 | 1.45444E-05 | 2.90888E-05 | 0.000014544409850996 | 0.000029088819699 | 0.000014544409847920 | 8586.35 | 8586.35 | 0.00 | 0.00 |  |
| 3 | 6371009 | 0.0008333333 | 0.000014544409851509 | 0.0016666666 | 0.0024999999 | 2.90888E-05 | 4.36332E-05 | 0.000029088819698917 | 0.000043633229541 | 0.000014544409841767 | 8586.35 | 8586.35 | 0.00 | 0.00 |  |
| 4 | 6371009 | 0.0008333333 | 0.000014544409851509 | 0.0024999999 | 0.0033333332 | 4.36332E-05 | 5.81776E-05 | 0.000043633229540683 | 0.000058177639373 | 0.000014544409832537 | 8586.35 | 8586.35 | 0.00 | 0.00 |  |
| 5 | 6371009 | 0.0008333333 | 0.000014544409851509 | 0.0033333332 | 0.0041666665 | 5.81776E-05 | 7.2722E-05 | 0.000058177639373220 | 0.000072722049193 | 0.000014544409820230 | 8586.35 | 8586.35 | 0.00 | 0.00 |  |
| 6 | 6371009 | 0.0008333333 | 0.000014544409851509 | 0.0041666665 | 0.0049999998 | 7.2722E-05 | 8.72665E-05 | 0.000072722049193449 | 0.000087266458998 | 0.000014544409804846 | 8586.35 | 8586.35 | 0.00 | 0.00 |  |
| 7 | 6371009 | 0.0008333333 | 0.000014544409851509 | 0.0049999998 | 0.0058333331 | 8.72665E-05 | 0.000101811 | 0.000087266458998295 | 0.000101810868785 | 0.000014544409786386 | 8586.35 | 8586.35 | 0.00 | 0.00 |  |
| 8 | 6371009 | 0.0008333333 | 0.000014544409851509 | 0.0058333331 | 0.0066666664 | 0.000101811 | 0.000116355 | 0.000101810868784682 | 0.000116355278550 | 0.000014544409764849 | 8586.35 | 8586.35 | 0.00 | 0.00 |  |
| 9 | 6371009 | 0.0008333333 | 0.000014544409851509 | 0.0066666664 | 0.0074999997 | 0.000116355 | 0.0001309 | 0.000116355278549530 | 0.000130899688290 | 0.000014544409740235 | 8586.35 | 8586.35 | 0.00 | 0.00 |  |
| 10 | 6371009 | 0.0008333333 | 0.000014544409851509 | 0.0074999997 | 0.0083333330 | 0.0001309 | 0.000145444 | 0.000130899688289765 | 0.000145444098002 | 0.000014544409712544 | 8586.35 | 8586.35 | 0.00 | 0.00 |  |
| 11 | 6371009 | 0.0008333333 | 0.000014544409855096 | 0.0083333330 | 0.0091666663 | 0.000145444 | 0.000159989 | 0.000145444098002309 | 0.000159988507684 | 0.000014544409681777 | 8586.35 | 8586.35 | 0.00 | 0.00 |  |
| 12 | 6371009 | 0.0008333333 | 0.000014544409851509 | 0.0091666663 | 0.0099999996 | 0.000159989 | 0.000174533 | 0.000159988507684087 | 0.000174532917332 | 0.000014544409647933 | 8586.35 | 8586.35 | 0.00 | 0.00 |  |
| 13 | 6371009 | 0.0008333333 | 0.000014544409851509 | 0.0099999996 | 0.0108333329 | 0.000174533 | 0.000189077 | 0.000174532917332020 | 0.000189077326943 | 0.000014544409611013 | 8586.35 | 8586.35 | 0.00 | 0.00 |  |
| 14 | 6371009 | 0.0008333333 | 0.000014544409851509 | 0.0108333329 | 0.0116666662 | 0.000189077 | 0.000203622 | 0.000189077326943032 | 0.000203621736514 | 0.000014544409571015 | 8586.35 | 8586.35 | 0.00 | 0.00 |  |
| 15 | 6371009 | 0.0008333333 | 0.000014544409851509 | 0.0116666662 | 0.0124999995 | 0.000203622 | 0.000218166 | 0.000203621736514047 | 0.000218166146042 | 0.000014544409527941 | 8586.35 | 8586.35 | 0.00 | 0.00 |  |
| 16 | 6371009 | 0.0008333333 | 0.000014544409851509 | 0.0124999995 | 0.0133333328 | 0.000218166 | 0.000232711 | 0.000218166146041988 | 0.000232710555524 | 0.000014544409481790 | 8586.35 | 8586.35 | 0.00 | 0.00 |  |
| 17 | 6371009 | 0.0008333333 | 0.000014544409851509 | 0.0133333328 | 0.0141666661 | 0.000232711 | 0.000247255 | 0.000232710555523778 | 0.000247254964956 | 0.000014544409432563 | 8586.35 | 8586.35 | 0.00 | 0.00 |  |
| 18 | 6371009 | 0.0008333333 | 0.000014544409851509 | 0.0141666661 | 0.0149999994 | 0.000247255 | 0.000261799 | 0.000247254964956341 | 0.000261799374337 | 0.000014544409380258 | 8586.35 | 8586.35 | 0.00 | 0.00 |  |
| 19 | 6371009 | 0.0008333333 | 0.000014544409851509 | 0.0149999994 | 0.0158333327 | 0.000261799 | 0.000276344 | 0.000261799374336599 | 0.000276343783661 | 0.000014544409324877 | 8586.35 | 8586.35 | 0.00 | 0.00 |  |
| 20 | 6371009 | 0.0008333333 | 0.000014544409851509 | 0.0158333327 | 0.0166666660 | 0.000276344 | 0.000290888 | 0.000276343783661477 | 0.000290888192928 | 0.000014544409266420 | 8586.35 | 8586.35 | 0.00 | 0.00 |  |
| **60 arc-second resolution (containing 400 pixels having a resolution of 3 arc-seconds)** | | | | | | | | | | | | | | | |
| 1 | 6371009 | **0.0166666660** | 0.000290888197030193 | 0.0000000000 | 0.0166666660 | 0 | 0.000290888 | 0.000000000000000000 | 0.000290888192928 | 0.000290888192927896 | 3434540 | 8586.35 |  |  |  |

**Supplementary Table 2a. Cell surface area calculation: Grid resolution induced error near the equator.**

**In a comparison of calculated area values in the 60 arc-second grid with those calculated for a small number of cells in a 3 arc-second grid, mean absolute error (MAE) in cell area is zero at low latitudes. Latitude increases down the table, thus cell area decreases - as this represents movement away from the equator towards the poles. Pi is defined as 3.14159265358979 for the purpose of this comparison. Error values expressed as zero are < ε. ε is 0.00.**

| **3 arc-second resolution** | | | | | | | | | | | | | | | |
| --- | --- | --- | --- | --- | --- | --- | --- | --- | --- | --- | --- | --- | --- | --- | --- |
| Px no. from the tile bottom | R2 (m) | CellSizeDeg | CellSizeRad | LowerLat | UpperLat | LowerLatRad | UpperLatRad | SinLowLatRad | SinUppLatRad | SinUpp-SinLow | Px AREA (m2) 3as resolution | Px AREA (m2) 60as resolution | Error | ABS (Error) | **MAE** |
| 1 | 6371009 | 0.0008333333 | 0.000014544409851509 | 51.8366606584 | 51.8374939917 | 0.904720402 | 0.904734946 | 0.786252420394722000 | 0.786261407381513 | 0.000008986986790749 | 5305.50 | 5304.57 | 0.93 | 0.93 | **0.49** |
| 2 | 6371009 | 0.0008333333 | 0.000014544409851509 | 51.8374939917 | 51.8383273250 | 0.904734946 | 0.904749491 | 0.786261407381513000 | 0.786270394201978 | 0.000008986820465018 | 5305.41 | 5304.57 | 0.83 | 0.83 |  |
| 3 | 6371009 | 0.0008333333 | 0.000014544409851509 | 51.8383273250 | 51.8391606583 | 0.904749491 | 0.904764035 | 0.786270394201978000 | 0.786279380856116 | 0.000008986654137511 | 5305.31 | 5304.57 | 0.74 | 0.74 |  |
| 4 | 6371009 | 0.0008333333 | 0.000014544409851509 | 51.8391606583 | 51.8399939916 | 0.904764035 | 0.904778579 | 0.786279380856116000 | 0.786288367343924 | 0.000008986487808116 | 5305.21 | 5304.57 | 0.64 | 0.64 |  |
| 5 | 6371009 | 0.0008333333 | 0.000014544409851509 | 51.8399939916 | 51.8408273249 | 0.904778579 | 0.904793124 | 0.786288367343924000 | 0.786297353665400 | 0.000008986321476723 | 5305.11 | 5304.57 | 0.54 | 0.54 |  |
| 6 | 6371009 | 0.0008333333 | 0.000014544409851509 | 51.8408273249 | 51.8416606582 | 0.904793124 | 0.904807668 | 0.786297353665400000 | 0.786306339820544 | 0.000008986155143553 | 5305.01 | 5304.57 | 0.44 | 0.44 |  |
| 7 | 6371009 | 0.0008333333 | 0.000014544409851509 | 51.8416606582 | 51.8424939915 | 0.904807668 | 0.904822213 | 0.786306339820544000 | 0.786315325809352 | 0.000008985988808496 | 5304.91 | 5304.57 | 0.34 | 0.34 |  |
| 8 | 6371009 | 0.0008333333 | 0.000014544409851509 | 51.8424939915 | 51.8433273248 | 0.904822213 | 0.904836757 | 0.786315325809352000 | 0.786324311631824 | 0.000008985822471330 | 5304.82 | 5304.57 | 0.25 | 0.25 |  |
| 9 | 6371009 | 0.0008333333 | 0.000014544409851509 | 51.8433273248 | 51.8441606581 | 0.904836757 | 0.904851301 | 0.786324311631824000 | 0.786333297287956 | 0.000008985656132388 | 5304.72 | 5304.57 | 0.15 | 0.15 |  |
| 10 | 6371009 | 0.0008333333 | 0.000014544409851509 | 51.8441606581 | 51.8449939914 | 0.904851301 | 0.904865846 | 0.786333297287956000 | 0.786342282777748 | 0.000008985489791669 | 5304.62 | 5304.57 | 0.05 | 0.05 |  |
| 11 | 6371009 | 0.0008333333 | 0.000014544409851509 | 51.8449939914 | 51.8458273247 | 0.904865846 | 0.90488039 | 0.786342282777748000 | 0.786351268101197 | 0.000008985323448840 | 5304.52 | 5304.57 | -0.05 | 0.05 |  |
| 12 | 6371009 | 0.0008333333 | 0.000014544409851509 | 51.8458273247 | 51.8466606580 | 0.90488039 | 0.904894935 | 0.786351268101197000 | 0.786360253258301 | 0.000008985157104235 | 5304.42 | 5304.57 | -0.15 | 0.15 |  |
| 13 | 6371009 | 0.0008333333 | 0.000014544409851509 | 51.8466606580 | 51.8474939913 | 0.904894935 | 0.904909479 | 0.786360253258301000 | 0.786369238249059 | 0.000008984990757743 | 5304.33 | 5304.57 | -0.25 | 0.25 |  |
| 14 | 6371009 | 0.0008333333 | 0.000014544409851509 | 51.8474939913 | 51.8483273246 | 0.904909479 | 0.904924023 | 0.786369238249059000 | 0.786378223073468 | 0.000008984824409253 | 5304.23 | 5304.57 | -0.34 | 0.34 |  |
| 15 | 6371009 | 0.0008333333 | 0.000014544409851509 | 51.8483273246 | 51.8491606579 | 0.904924023 | 0.904938568 | 0.786378223073468000 | 0.786387207731527 | 0.000008984658058986 | 5304.13 | 5304.57 | -0.44 | 0.44 |  |
| 16 | 6371009 | 0.0008333333 | 0.000014544409851509 | 51.8491606579 | 51.8499939912 | 0.904938568 | 0.904953112 | 0.786387207731527000 | 0.786396192223234 | 0.000008984491706721 | 5304.03 | 5304.57 | -0.54 | 0.54 |  |
| 17 | 6371009 | 0.0008333333 | 0.000014544409851509 | 51.8499939912 | 51.8508273245 | 0.904953112 | 0.904967657 | 0.786396192223234000 | 0.786405176548586 | 0.000008984325352457 | 5303.93 | 5304.57 | -0.64 | 0.64 |  |
| 18 | 6371009 | 0.0008333333 | 0.000014544409851509 | 51.8508273245 | 51.8516606578 | 0.904967657 | 0.904982201 | 0.786405176548586000 | 0.786414160707583 | 0.000008984158996528 | 5303.83 | 5304.57 | -0.74 | 0.74 |  |
| 19 | 6371009 | 0.0008333333 | 0.000014544409851509 | 51.8516606578 | 51.8524939911 | 0.904982201 | 0.904996746 | 0.786414160707583000 | 0.786423144700221 | 0.000008983992638600 | 5303.74 | 5304.57 | -0.83 | 0.83 |  |
| 20 | 6371009 | 0.0008333333 | 0.000014544409851509 | 51.8524939911 | 51.8533273244 | 0.904996746 | 0.90501129 | 0.786423144700221000 | 0.786432128526500 | 0.000008983826278786 | 5303.64 | 5304.57 | -0.93 | 0.93 |  |
| **60 arc-second resolution (containing 400 pixels having a resolution of 3 arc-seconds)** | | | | | | | | | | | | | | | |
| 1 | 6371009 | **0.0166666660** | 0.000290888197030193 | 51.8366606584 | 51.8533273244 | 0.904720402 | 0.90501129 | 0.786252420394722000 | 0.786432128526500 | 0.000179708131777367 | 2121828 | 5304.57 |  |  |  |

**Supplementary Table 2b. Cell surface area calculation: Grid resolution induced error at mid latitudes.**

**In a comparison of calculated area values in the 60 arc-second grid with those calculated for a small number of cells in a 3 arc-second grid, mean absolute error (MAE) in cell area is 0.49 at middle latitudes. Latitude increases down the table, thus cell area decreases - as this represents movement away from the equator towards the poles. Pi is defined as 3.14159265358979 for the purpose of this comparison.**

| **3 arc-second resolution** | | | | | | | | | | | | | | | |
| --- | --- | --- | --- | --- | --- | --- | --- | --- | --- | --- | --- | --- | --- | --- | --- |
| Px no. from the tile bottom | R2 (m) | CellSizeDeg | CellSizeRad | LowerLat | UpperLat | LowerLatRad | UpperLatRad | SinLowLatRad | SinUppLatRad | SinUpp-SinLow | Px AREA (m2) 3as resolution | Px AREA (m2) 60as resolution | Error | ABS (Error) | **MAE** |
| 1 | 6371009 | 0.0008333333 | 0.000014544409851509 | 89.9833333340 | 89.9841666673 | 1.570505439 | 1.570519983 | 0.999999957692029000 | 0.999999961817056 | 0.000000004125027098 | 2.44 | 1.25 | 1.19 | 1.19 | **0.62** |
| 2 | 6371009 | 0.0008333333 | 0.000014544409851509 | 89.9841666673 | 89.9850000006 | 1.570519983 | 1.570534527 | 0.999999961817056000 | 0.999999965730543 | 0.000000003913487312 | 2.31 | 1.25 | 1.06 | 1.06 |  |
| 3 | 6371009 | 0.0008333333 | 0.000014544409851509 | 89.9850000006 | 89.9858333339 | 1.570534527 | 1.570549072 | 0.999999965730543000 | 0.999999969432491 | 0.000000003701947526 | 2.19 | 1.25 | 0.94 | 0.94 |  |
| 4 | 6371009 | 0.0008333333 | 0.000014544409851509 | 89.9858333339 | 89.9866666672 | 1.570549072 | 1.570563616 | 0.999999969432491000 | 0.999999972922898 | 0.000000003490407630 | 2.06 | 1.25 | 0.81 | 0.81 |  |
| 5 | 6371009 | 0.0008333333 | 0.000014544409851509 | 89.9866666672 | 89.9875000005 | 1.570563616 | 1.570578161 | 0.999999972922898000 | 0.999999976201766 | 0.000000003278867733 | 1.94 | 1.25 | 0.69 | 0.69 |  |
| 6 | 6371009 | 0.0008333333 | 0.000014544409851509 | 89.9875000005 | 89.9883333338 | 1.570578161 | 1.570592705 | 0.999999976201766000 | 0.999999979269094 | 0.000000003067327947 | 1.81 | 1.25 | 0.56 | 0.56 |  |
| 7 | 6371009 | 0.0008333333 | 0.000014544409851509 | 89.9883333338 | 89.9891666671 | 1.570592705 | 1.570607249 | 0.999999979269094000 | 0.999999982124882 | 0.000000002855788050 | 1.69 | 1.25 | 0.44 | 0.44 |  |
| 8 | 6371009 | 0.0008333333 | 0.000014544409851509 | 89.9891666671 | 89.9900000004 | 1.570607249 | 1.570621794 | 0.999999982124882000 | 0.999999984769130 | 0.000000002644248154 | 1.56 | 1.25 | 0.31 | 0.31 |  |
| 9 | 6371009 | 0.0008333333 | 0.000014544409851509 | 89.9900000004 | 89.9908333337 | 1.570621794 | 1.570636338 | 0.999999984769130000 | 0.999999987201839 | 0.000000002432708368 | 1.44 | 1.25 | 0.19 | 0.19 |  |
| 10 | 6371009 | 0.0008333333 | 0.000014544409851509 | 89.9908333337 | 89.9916666670 | 1.570636338 | 1.570650883 | 0.999999987201839000 | 0.999999989423007 | 0.000000002221168582 | 1.31 | 1.25 | 0.06 | 0.06 |  |
| 11 | 6371009 | 0.0008333333 | 0.000014544409851509 | 89.9916666670 | 89.9925000003 | 1.570650883 | 1.570665427 | 0.999999989423007000 | 0.999999991432636 | 0.000000002009628575 | 1.19 | 1.25 | -0.06 | 0.06 |  |
| 12 | 6371009 | 0.0008333333 | 0.000014544409851509 | 89.9925000003 | 89.9933333336 | 1.570665427 | 1.570679972 | 0.999999991432636000 | 0.999999993230725 | 0.000000001798088789 | 1.06 | 1.25 | -0.19 | 0.19 |  |
| 13 | 6371009 | 0.0008333333 | 0.000014544409851509 | 89.9933333336 | 89.9941666669 | 1.570679972 | 1.570694516 | 0.999999993230725000 | 0.999999994817274 | 0.000000001586549003 | 0.94 | 1.25 | -0.31 | 0.31 |  |
| 14 | 6371009 | 0.0008333333 | 0.000014544409851509 | 89.9941666669 | 89.9950000002 | 1.570694516 | 1.57070906 | 0.999999994817274000 | 0.999999996192283 | 0.000000001375008996 | 0.81 | 1.25 | -0.44 | 0.44 |  |
| 15 | 6371009 | 0.0008333333 | 0.000014544409851509 | 89.9950000002 | 89.9958333335 | 1.57070906 | 1.570723605 | 0.999999996192283000 | 0.999999997355752 | 0.000000001163469210 | 0.69 | 1.25 | -0.56 | 0.56 |  |
| 16 | 6371009 | 0.0008333333 | 0.000014544409851509 | 89.9958333335 | 89.9966666668 | 1.570723605 | 1.570738149 | 0.999999997355752000 | 0.999999998307681 | 0.000000000951929424 | 0.56 | 1.25 | -0.69 | 0.69 |  |
| 17 | 6371009 | 0.0008333333 | 0.000014544409851509 | 89.9966666668 | 89.9975000001 | 1.570738149 | 1.570752694 | 0.999999998307681000 | 0.999999999048071 | 0.000000000740389527 | 0.44 | 1.25 | -0.81 | 0.81 |  |
| 18 | 6371009 | 0.0008333333 | 0.000014544409851509 | 89.9975000001 | 89.9983333334 | 1.570752694 | 1.570767238 | 0.999999999048071000 | 0.999999999576920 | 0.000000000528849631 | 0.31 | 1.25 | -0.94 | 0.94 |  |
| 19 | 6371009 | 0.0008333333 | 0.000014544409851509 | 89.9983333334 | 89.9991666667 | 1.570767238 | 1.570781782 | 0.999999999576920000 | 0.999999999894230 | 0.000000000317309734 | 0.19 | 1.25 | -1.06 | 1.06 |  |
| 20 | 6371009 | 0.0008333333 | 0.000014544409851509 | 89.9991666667 | 90.0000000000 | 1.570781782 | 1.570796327 | 0.999999999894230000 | 1.000000000000000 | 0.000000000105769948 | 0.06 | 1.25 | -1.19 | 1.19 |  |
| **60 arc-second resolution (containing 400 pixels having a resolution of 3 arc-seconds)** | | | | | | | | | | | | | | | |
| 1 | 6371009 | **0.0166666660** | 0.000290888197030193 | 89.9833333340 | 90.0000000000 | 1.570505439 | 1.570796327 | 0.999999957692029000 | 1.000000000000000 | 0.000000042307971237 | 500 | 1.25 |  |  |  |

**Supplementary Table 2c. Cell surface area calculation: Grid resolution induced error near the poles.**

**In a comparison of calculated area values in the 60 arc-second grid with those calculated for a small number of cells in a 3 arc-second grid, mean absolute error (MAE) in cell area is 0.62 at high latitudes. Latitude increases down the table, thus cell area decreases - as this represents movement away from the equator towards the poles. Pi is defined as 3.14159265358979 for the purpose of this comparison.**
